# Does the traffic light protocol capture dose deviations? A longitudinal CBCT-based dose-monitoring study in adaptive RT for LA-NSCLC^☆^

**DOI:** 10.1016/j.tipsro.2026.100427

**Published:** 2026-08-14

**Authors:** Dylan Callens, Emma Van Riet, Jan Verstraete, Patrick Berkovic, Maarten Lambrecht, Wouter Crijns

**Affiliations:** a Laboratory of Experimental Radiotherapy, KU Leuven, Leuven, Belgium; b Department of Radiation Oncology, UZ Leuven, Leuven, Belgium; c Leuven Cancer Institute (LKI), Leuven, Belgium

**Keywords:** Dose monitoring, Automation, CBCT-dose calculation, ART

## Abstract

**Introduction:**

Traffic light protocols (TLP) standardize IGRT by reporting anatomical alterations presumed to proxy dosimetric change, yet this assumption is rarely verified. We therefore characterize the daily delivered dose and its longitudinal evolution during adaptive radiotherapy for LA-NSCLC and quantify how TLP reporting relates to dosimetric alteration.

**Materials and methods:**

Twenty-two patients with LA-NSCLC enrolled in a prospective trial (604 fractions, mid-treatment adaptation) underwent multi-task automated daily CBCT-based dose monitoring. For each fraction, the delivered dose and the accumulated dose were compared with the reference plan utilizing the existing dose-volume constraints. Longitudinal dose evolution and TLP-code associations were analyzed with piecewise linear mixed-effects models, with significance based on BH-FDR-adjusted q-values (q < 0.10).

**Results:**

Daily delivered dose respected planning constraints (median differences within ±3%) but varied between patients. At the population level, only lung V20Gy showed a deviation rate from start (β = 0.082%-points/day; q = 0.044). The deviation rate after plan-adaptation was not significant. Population deviation rates were largely dominated by inter-patient variability. Three DVH-parameters were significantly associated with TLP-codes. Code orange for tumour alterations was associated with PTVp V95% reduction as well as spinal canal D0.03cc increase of respectively −1.73%-points (q = 0.011) and 2.15%-points (q = 0.011). Code orange for spinal-canal alterations was also associated with PTVp V95%-reduction (β = −2.03%-points, q = 0.002). Code orange for lungs showed no significant association with any dose constraint after adjustment for days on treatment.

**Conclusion:**

TLP-code orange reporting is a partial proxy for PTV-coverage loss but an unreliable proxy for organ-at-risk dose. Dose deviation rates during treatment are patient specific. This highlights the need for continuous daily dose monitoring to inform ART-decisions during treatment.

## Highlights

• Dose deviation rates across treatment are patient-specific.
• TLP-code orange partially flagged PTV coverage loss.
• TLP reporting is an unreliable proxy for organ-at-risk dose alterations.
• Daily CBCT dose monitoring adds objective dose data to subjective TLP review.

## Introduction

Patients with locally advanced non-small-cell lung cancer (LA-NSCLC) are treated with chemoradiotherapy delivered over several weeks [1], [2]. During these long treatment courses, both transient and systematic anatomical alterations can occur. These alterations include day-to-day variations such as diaphragm position, pleural effusion fluctuations, patient setup variability as well as more gradual processes such as tumour regression, mediastinal shift or lung changes [3], [4], [5], [6]. Daily cone-beam CT (CBCT) imaging allows radiation therapists (RTTs) to identify these alterations and, where possible, correct for them through image registration and subsequent couch corrections.

In routine clinical practice, this image registration process is frequently supported by an anatomy-based traffic light protocol (TLP) [6], [7], [8]. Such TLPs have been instrumental in the broad implementation of CBCT-based IGRT as it standardizes the image registration process and functions as a structured, colour-coded communication tool between disciplines. This fraction-wise communication is triggered by code ‘orange’ or ‘red’ and may result in plan-adaptations [7], [9].

TLPs rely primarily on visual anatomical assessment [10]. As a result, it often remains unclear to what extent a reported anatomical alteration can be seen as proxy for dosimetric alterations. The lack of this insight into the delivered dose across fractions introduces ambiguity into decisions on subsequent steps including the choice to initiate plan adaptation [11], [12]. The maturation of CBCT-based dose calculation and automation has made it possible to address this uncertainty [13], [14], [15], as demonstrated using daily delivered dose monitoring in a recent dose-escalation trial [16].

This study evaluates daily CBCT-based dose monitoring in patients with LA-NSCLC through an automated workflow and quantifies its association with anatomy-based traffic light protocol reporting using piecewise linear mixed-effects modelling. The aims are to: (i) characterize delivered dose and its longitudinal evolution before and after a scheduled mid-treatment adaptation; and (ii) quantify the association between daily anatomical TLP reporting and daily constraint-specific delivered dose.

## Materials and methods

### Patient cohort

Twenty-two patients with LA-NSCLC enrolled in the prospective mid-treatment adaptive radiotherapy trial ECLAIR (NCT07259447) were included, treated between July 2024 and March 2026. The dataset entails 604 fractions. ECLAIR aims to collect prospective benchmark data on mid-treatment CT-based ART, which will subsequently be used to evaluate the feasibility, time efficiency and contouring and planning quality of automated CBCT-based ART. The present analysis addresses a secondary objective of ECLAIR, providing a dosimetric baseline of current clinical practice.

Patient characteristics are presented in Supplementary Table 1. Twelve patients received concurrent chemoradiotherapy (30–33 fractions of 2.0 Gy), seven received sequential chemoradiotherapy (17–24 fractions of 2.75 Gy), two received induction chemotherapy followed by radiotherapy (25 fractions of 1.8 Gy), and one received radiotherapy alone (22 fractions of 2.75 Gy). All patients underwent a protocol-scheduled mid-treatment plan adaptation based on a repeated planning-CT, independently of the daily TLP codes.

Target volumes were delineated on the mean intensity projection of a 4DCT (Somatom Definition Edge or Drive, Siemens Healthineers, Munich, Germany) in accordance with international guidelines [17], [18]. The internal gross tumour volume (iGTV) was expanded by 5 mm to the clinical target volume (CTV) and by a further isotropic 7 mm to the planning target volume (PTV). IMRT plans of nine 6 MV beams were generated in Eclipse (Varian, a Siemens Healthineers company, Palo Alto, CA, USA) using RapidPlan. The dose was calculated with Acuros 18.1. Plans were evaluated utilizing our clinical dose-volume constraints (see further). Treatment was delivered on a Halcyon linac.

### TLP workflow

Of the cohort, ten patients were scanned in free breathing using Halcyon 4.0 CBCT (standard thorax acquisition, standard reconstruction). Twelve patients underwent daily HyperSight imaging in free breathing (60s acquisition, iterative reconstruction). This allocation was not randomized but determined by the clinical implementation of HyperSight imaging on all our linacs during the prospective trial. Previous work demonstrated comparable image quality and dosimetric outcome between both acquisition and reconstruction approaches in lung, supporting their generalized use in this analysis [13], [19].

TLP decision guidance was based on our TLP described in [10], and illustrated in Suppl. Fig. 1, a four-step process: (1) mediastinal rigid 3D-registration; (2) tumour coverage review; (3) spinal canal review; and (4) lung review, each scored green, orange, or red against domain-specific criteria.

For tumour coverage, code orange was assigned when the tumour extended beyond the iGTV + 2 mm evaluation contour while remaining within the 90% prescription isodose (V90), and code red when it extended beyond the 90% isodose.

For the spinal canal, code orange was assigned when positional or anatomical change displaced the spinal canal beyond its planning-risk-volume margin while remaining outside the 50 Gy tolerance isodose, and code red when it was displaced into the 50 Gy tolerance isodose.

For the lungs, code orange reflected in- or exsufflation with associated lung volume or density change.

A code green indicates standard continuation. A code orange functions as an early warning: the fraction is prompted earlier review by the treating physician in place of the routine weekly review of a single arbitrary fraction. Based on this early warning, in severe anatomical alterations, a recalculation on the CBCT is requested. A code red requires online physician intervention before the fraction can continue.

In this study, all treatment fractions were TLP-coded retrospectively by one of the authors (DC). This approach was adopted because our previous work demonstrated that underreporting occurs in clinical practice [10]. Each reported code orange/red was documented with the specific reason for it.

### CBCT-based dose calculation workflow

Daily delivered dose calculation on CBCT was multi-task automated (level 2) [20] in MIM Software (MIM Software Inc., Cleveland, OH, USA) using the ART Assist platform (Suppl. Fig. 2). CBCT images were manually imported into a predefined worklist, after which the platform propagated each CBCT through the workflow using rule-based automation.

Within this workflow, each CBCT was rigidly registered to the corresponding planning CT and extended cranio-caudally using planning CT slices to include the full thorax required for dose calculation. Organs-at-risk (OAR) were then propagated from the planning CT to the CBCT using deformable image registration (DIR), whereas target structures were transferred rigidly, in accordance with adaptive radiotherapy practice [21]. Propagated structures underwent ad-hoc visual review on a random subset of fractions per patient.

Dose calculation on the CBCT was performed using SureCalc® (SciMoCa®), a Monte Carlo (MC) dose engine, with a statistical uncertainty of 1% over the high-dose region and a dose grid resolution of 2.5 mm. CBCT intensities were converted to density using the HU-to-density calibration curve implemented in MIM. The delivered dose was scaled to the total number of prescribed fractions. In addition, each fraction's dose distribution was non-rigidly deformed back to the pre-treatment planning CT for accumulated dose assessment.

### Data analysis plan

#### Reported codes

Code orange/red reporting was counted descriptively for all fractions of the treatment course. Progress in the course was reported relatively with respect to the total course length (0–100%) and divided into 20 bins of 5%, allowing code orange/red fractions to be reported across patients on a common scale.

#### Automation of fraction-wise dose calculation on CBCT

The analysis of automation focused on time efficiency. The multi-task automated fraction-wise dose calculation pipeline was evaluated by logging processing times for each CBCT-dose calculation [20].

#### Daily delivered and accumulated dose analyses per patient

Dose-volume histogram (DVH)-parameters were collected per fraction, further referred to as the daily delivered dose. Subsequently, the delivered dose was compared with the planned dose on the pre-treatment or mid-treatment planning-CT, depending on the treatment phase. These reference plans were recalculated with the SureCalc algorithm to avoid bias [22]. The comparison was expressed as relative difference between daily delivered dose and planned dose (ΔD and ΔV, [%]). Additionally, accumulated doses were compared to the pre-treatment plan.

The dose constraints followed the clinical protocol DVH-parameters: CTVp/n Dmean and V95%, PTVp/n D99% and V95%, Brachial Plexi D0.03cc, Contralateral Lung V5Gy, Esophagus_05 D0.03cc, Heart D0.03cc, Heart Dmean, Lungs Dmean, Lungs V20Gy, Lungs V30Gy, Lungs V5Gy, Mediastinal Envelope_05 D0.03cc and Spinal Canal D0.03cc. The ‘_0x’ suffix denotes the planning-risk-volume margin.

Inferential analysis was restricted to DVH-parameters selected for target proximity and relevance to the TLP framework. CTVp/n and PTVp/n V95% reflect the coverage assessed at TLP review. Lung V20Gy was selected as a clinically relevant lung-dose constraint that may be influenced by changes in lung volume and density during treatment [23]. Spinal Canal D0.03cc is sensitive to matching, given the canal's proximity to the V50Gy region. Mediastinal Envelope_05, Heart, and Esophagus_05 D0.03cc are retained because mediastinal matching may shift the maximum dose.

#### Association between delivered doses, longitudinal evolution and TLP reporting

**Days on treatment vs. delivered dose differences**

To characterize the evolution of delivered dose differences in function of the days on treatment, piecewise linear mixed-effects models were fit using maximum likelihood estimation (Equation (1)). For each dose constraint a separate model was fitted. The models estimated the dose or volume (ΔD or ΔV) deviation rate before (β₁ [%-point/day]) and after (β₃ [%-point/day]) the plan-adaptation, with an expected immediate dose correction on the time of the plan-adaptation (β₂ [%]). The deviation rates were modelled longitudinally in function of calendar days, thereby accounting for weekends and treatment interruptions.

The models included a mixed-effects structure to estimate ΔD per fraction, while accounting for both inter-and intra-patient (n_patients_ = 22) variability across fractions (n_fractions_ = 604). A maximal random-effects structure supported by the data was used, including patient-specific random intercepts and random slopes for all three phases (before plan-adaptation, adaptation, post-adaptation). This allowed each patient to have their own ΔD/V baseline level, as well their own ΔD/V dose drift over days on treatment, modelled per phase.

Maximum likelihood estimation selects the parameter values that make the observed data most plausible. An illustration of the statistical analysis can be found in Suppl. Fig. 3.

To control false-positives from multiple testing, *p*-values were corrected using the Benjamini-Hochberg false discovery rate procedure (BH-FDR). Given the exploratory nature of this analysis, BH-FDR-adjusted q-values below 0.10 were considered statistically significant [24], [25].

(1) $$\mathrm{\Delta D}=\beta_{0}+\beta_{1}\cdot t_{\mathrm{pre}}+\beta_{2}\cdot \mathrm{post}+\beta_{3}\cdot t_{\mathrm{post}}+v_{1i}\cdot t_{\mathrm{pre},z}+u_{2i}\cdot \mathrm{post}+v_{3i}\cdot t_{\mathrm{post},z}+u_{0i}+\varepsilon$$

Equation (1). β₀: population mean dose deviation on the last day before adaptation. β₁: population deviation rate before adaptation (%-points/day). β₂: population immediate dose shift at adaptation. β₃: population deviation rate after adaptation (%-points/day). v₁ᵢ, u_i_, v₃ᵢ: patient-specific random effects on standardized t_pre, post, and t_post allowing each patient an individual pre-adaptation drift rate, adaptation-induced level shift, and post-adaptation drift rate. ε: residual fraction-level error. $t_{\mathrm{pre}}$ was set to 0 on the day before the adapted plan started and took negative values for earlier pre-adaptation days. postwas coded as 0 before adaptation and 1 from the start of the adapted plan onwards. $t_{\text{post}}$was set to 0 before adaptation and counted the number of calendar days since the start of the adapted plan thereafter.

To characterize inter-patient variability in the deviation rates, a separate ordinary least-squares line was fitted per patient. The resulting deviation rates observed (%-points/day) were summarized across patients by their range and standard deviation.

**TLP reporting vs. delivered dose differences**

To assess whether fractions reported as code orange were associated with additional delivered dose deviations after accounting for days on treatment, the longitudinal model (Equation (1)) was extended with reporting of code orange (Equation (2)). This code orange model was independently fit for each dose constraint and for each code orange type (tumour, spinal canal, lungs):

(2) $$\mathrm{\Delta D}=\beta_{0}+\beta_{1}\cdot t_{\mathrm{pre}}+\beta_{2}\cdot \mathrm{post}+\beta_{3}\cdot t_{\mathrm{post}}+\beta_{4}\cdot \mathrm{orange}+v_{1i}\cdot t_{\mathrm{pre},z}+u_{2i}\cdot \mathrm{post}+v_{3i}\cdot t_{\mathrm{post},z}+u_{0i}+\varepsilon$$

Equation (2). All terms retain the same interpretation as in Equation (1). β₄·orange is the mean dose deviation of a code orange reported fraction relative to a code green reported fraction at the same point in the treatment course, within the same patient, after accounting for days on treatment.

Code orange model (Equation (2)) was compared with the longitudinal model (Equation (1)) using a likelihood ratio test (LRT) and the change in Akaike Information Criterion (ΔAIC). AIC was calculated using the standard formula (AIC = -2log(L) + 2k), where L is the maximized model likelihood and k the number of estimated model parameters [26]. A significant LRT test (q < 0.10) combined with a decrease in AIC of more than two points, indicated that code orange reporting contributes information about delivered dose beyond the longitudinal treatment-time effects.

All analyses were carried out in Python (v3.10) using statsmodels package [27]. Model output was validated in R (v4.6).

## Results

### Reported TLP-codes

In total, 38% of fractions received a code orange, with the majority being for code orange lungs (Table 1). There were no fractions with a code red.

**Table 1:** Distribution of TLP flags.

	TLP_2026_
Total Fractions	604
Total Patients	22

	IGRT-RTT (DC)
Total patients with minimum 1 reported code	19 (86%)
Total patients with systematic error *(3 consecutive codes)*	15 (68%)
Total fractions with Code Orange	232 (38%)
Total fractions with Code Red	0 (0%)
Code Orange	
Tumour coverage (i.e. volume increase, shift)	58 (10%)
Spinal Canal (i.e. positioning/proximality to V50Gy)	48 (8%)
Lungs (i.e. insufflation/exsufflation, lung density changes, positioning errors)	187 (31%)

**Fig. 1:**
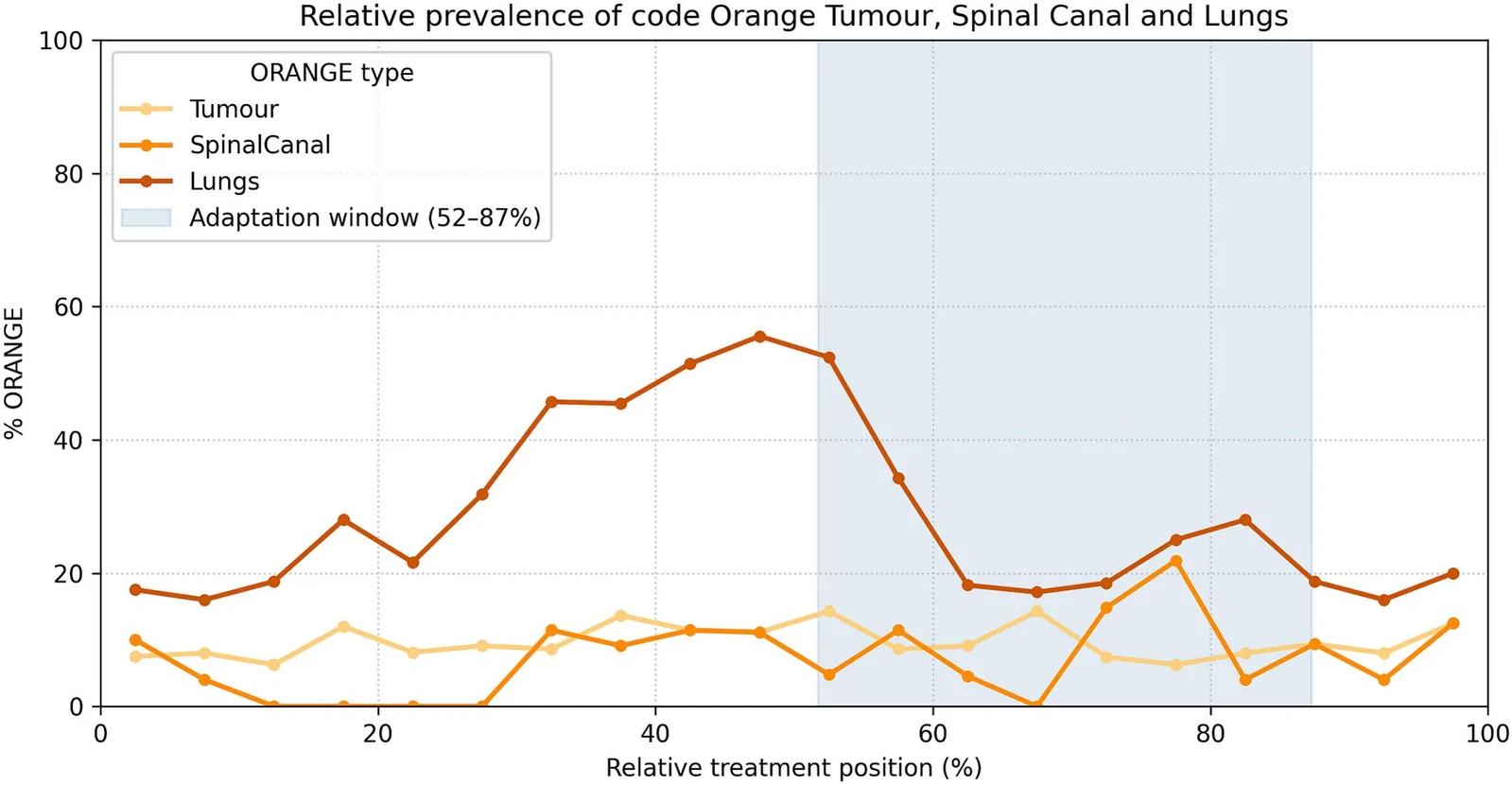
Code orange reported throughout the treatment, relative expressed compared to the total number of fractions. The plotted adaptation window is the 2.5th‐97.5th percentile of the patient-specific relative adaptation-start positions. Variations in this window will be reported in the ECLAIR primary analyses.

Code orange for tumour and spinal canal did not show a reporting trend and were assigned scattered throughout the course. In contrast, code orange for lungs showed an increasing trend before the population-wide adaptation window, followed by a clear reduction during it (Fig. 1).

### Automation of fraction-wise dose calculation on CBCT

On average, 5min20s was spent per automated CBCT-based dose calculation (SD = 43s), with a minimum of 3min14s and a maximum of 7min13s. Five fractions (0.8%) required unplanned manual continuation because of automation failure.

### Daily delivered and accumulated dose per patient

**Fig. 2:**
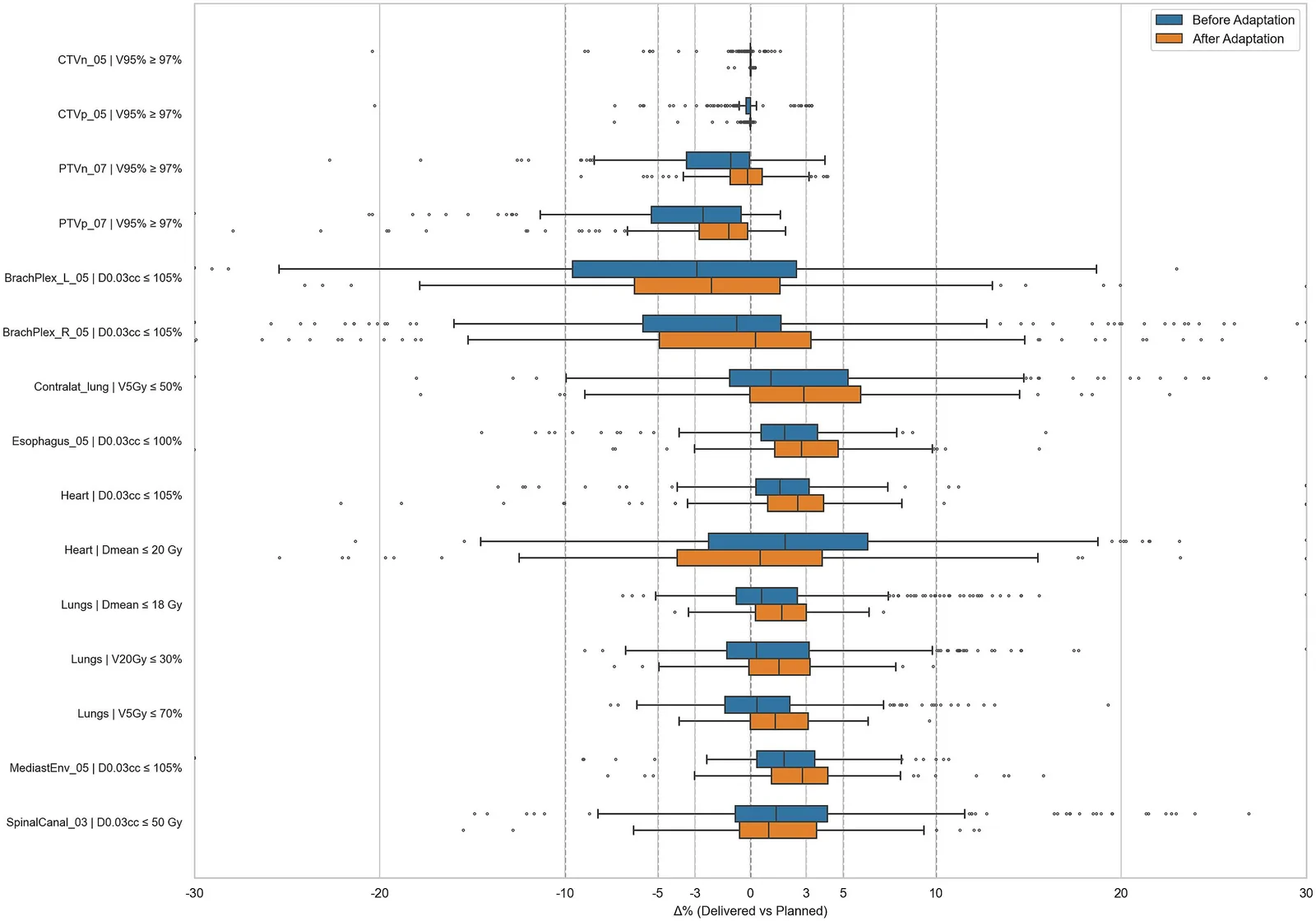
Relative dose differences, categorized before and after adaptation. For targets only V95% is shown; D99%, Dmean and Lungs V30Gy are reported in Table 2. An in-depth variance and variability analysis has been reported elsewhere [28].

The median daily delivered dose differences are within ±3% compared to the reference plan (Fig. 2). The brachial plexi showed the highest variability, most pronounced pre-adaptation (left IQR 12.1%, right 7.4%). Dose differences before versus after adaptation were not significantly different based on a Wilcoxon signed-rank test.

For the accumulated dose (Table 2), target coverage decreased. Mean PTVp and PTVn D99% decreased by −12.51% and −13.12%, relative to the pre-treatment plan. Mean V95% decreased by −5.62% for PTVp and −3.26% for PTVn.

**Table 2:** Dosimetric constraint adherence across pre-treatment planning, mid-treatment planning, and total accumulated dose. Pre-treatment violations indicate the number of patients constraint violation was accepted before treatment start. Mid-treatment violations are observed at the adaptation moment. Accumulated dose violations indicate whether pre-treatment violations persisted into the total accumulated delivered dose and whether new violations emerged in patients who were compliant at pre-treatment. N reflects the total number of patients for which the structure was available. NA, not applicable.

Structure	Dose- constraint	Pre-treatment dose ± SD [min; max]	Mean Accumulated ΔD % ± SD [min; max] *(delivered* vs *Pre)*	Pre-treatment violations, n/N (%)	*Mid-treatment violations, n/N (%)*	Accumulated dose violations *(delivered* vs *Pre)*
CTVn_05	Dmean ≥97%	100.43 ± 1.17 [98.20; 102.37]	1.48 ± 1.39 [−2.33; 4.28]	NA	*NA*	NA
	V95% ≥ 97%	99.73 ± 0.59 [98.84; 100.00]	0.11 ± 0.71 [−0.75; 1.67]	NA	*NA*	NA
CTVp_05	Dmean ≥97%	100.70 ± 1.25 [96.76; 103.70]	0.60 ± 1.00 [−1.19; 2.72]	NA	*NA*	NA
	V95% ≥ 97%	99.78 ± 0.70 [97.19; 100.00]	−0.95 ± 2.97 [−10.86; 2.26]	1/22 (5%)	*NA*	0/1 persistent +2 new
PTVn_07	D99% ≥ 90%	55.52 ± 4.26 [49.78; 63.83]	−13.12 ± 10.71 [−40.48; 0.94]	2/17 (12%)	*NA*	2/2 persistent +12 new
	V95% ≥ 97%	96.93 ± 2.51 [90.29; 99.57]	−3.26 ± 3.00 [−10.22; 1.84]	7/17 (42%)	*2/17 (12%)*	6/7 persistent +8 new
PTVp_07	D99% ≥ 90%	54.06 ± 6.26 [42.87; 62.76]	−12.51 ± 14.97 [−49.39; 13.71]	3/22 (14%)	*4/22 (18%)*	2/3 persistent +14 new
	V95% ≥ 97%	97.49 ± 2.80 [85.97; 99.99]	−5.62 ± 8.58 [−32.58; 11.81]	7/22 (32%)	*3/22 (14%)*	6/7 persistent +12 new
BrachPlex_L_05	D0.03cc ≤ 105%	13.24 ± 13.90 [0.74; 45.54]	−8.42 ± 12.34 [−29.70; 23.06]	NA	*NA*	NA
BrachPlex_R_05	D0.03cc ≤ 105%	29.08 ± 27.67 [0.82; 66.44]	−9.31 ± 11.47 [−32.82; 8.87]	1/17 (6%)	*NA*	0/1 persistent
Contralat_lung	V5Gy ≤ 50%	38.63 ± 24.82 [0.23; 89.16]	2.30 ± 10.48 [−22.06; 31.94]	5/21 (24%)	*5/22 (23%)*	5/5 persistent +1 new
Esophagus_05	D0.03cc ≤ 100%	56.02 ± 13.42 [19.08; 69.05]	−1.05 ± 5.78 [−19.36; 6.04]	15/22 (68%)	*16/22 (73%)*	15/15 persistent +1 new
Heart	D0.03cc ≤ 105%	53.60 ± 20.44 [1.22; 68.69]	2.60 ± 8.91 [−4.28; 38.34]	4/21 (19%)	*1/22 (5%)*	3/4 persistent +2 new
	Dmean ≤20Gy	9.40 ± 6.59 [0.37; 22.74]	2.41 ± 7.55 [−11.05; 17.16]	2/21 (9%)	*2/22 (9%)*	1/2 persistent
Lungs	Dmean ≤18Gy	12.53 ± 3.78 [6.13; 19.64]	0.64 ± 2.91 [−5.19; 7.01]	3/22 (14%)	*3/22 (14%)*	3/3 persistent
	V20Gy ≤ 30%	21.86 ± 7.35 [9.74; 35.28]	1.11 ± 6.08 [−9.67; 20.00]	4/22 (18%)	*3/22 (14%)*	4/4 persistent +1 new
	V30Gy ≤ 20%	14.68 ± 5.00 [6.63; 24.54]	−0.34 ± 5.63 [−12.22; 10.58]	4/22 (18%)	*4/22 (18%)*	3/4 persistent
	V5Gy ≤ 70%	51.17 ± 19.20 [20.65; 92.38]	1.33 ± 3.38 [−4.50; 8.13]	4/22 (18%)	*3/22 (14%)*	4/4 persistent
MediastEnv_05	D0.03cc ≤ 105%	59.20 ± 11.08 [20.56; 70.56]	−0.68 ± 2.32 [−5.47; 3.70]	8/20 (40%)	*12/22 (55%)*	7/8 persistent +2 new
SpinalCanal_03	D0.03cc ≤ 50Gy	39.53 ± 9.49 [13.47; 53.55]	−0.36 ± 5.57 [−12.44; 13.50]	3/22 (14%)	*2/22 (9%)*	3/3 persistent

The numerical suffix indicates the isotropic margin in millimetres used to derive the evaluated structure, including target-volume expansions and planning organ-at-risk volume margins.

For all organs-at-risk except the brachial plexi, accumulated dose deviations remained below Δ3% relative to the pre-treatment plan. The spinal canal showed pre-treatment D0.03cc constraint violations in three patients. Accumulated organ-at-risk doses were comparable to the pre-treatment planning doses.

Although comparable on average with the reference plan, accumulated dose differences showed large patient-specific variability. This was largest for target coverage (PTVp D99% −49.4% to 13.7%; PTVp V95% −32.6% to 11.8%) and, among organs-at-risk, for the heart (D0.03cc −4.3% to 38.3%), contralateral lung (V5Gy −22.1% to 31.9%) and brachial plexi (D0.03cc −32.8% to 23.1%).

### Longitudinal analysis of DVH parameters and TLP reporting

#### ΔD and ΔV-deviation rate throughout the ART course

**Fig. 3:**
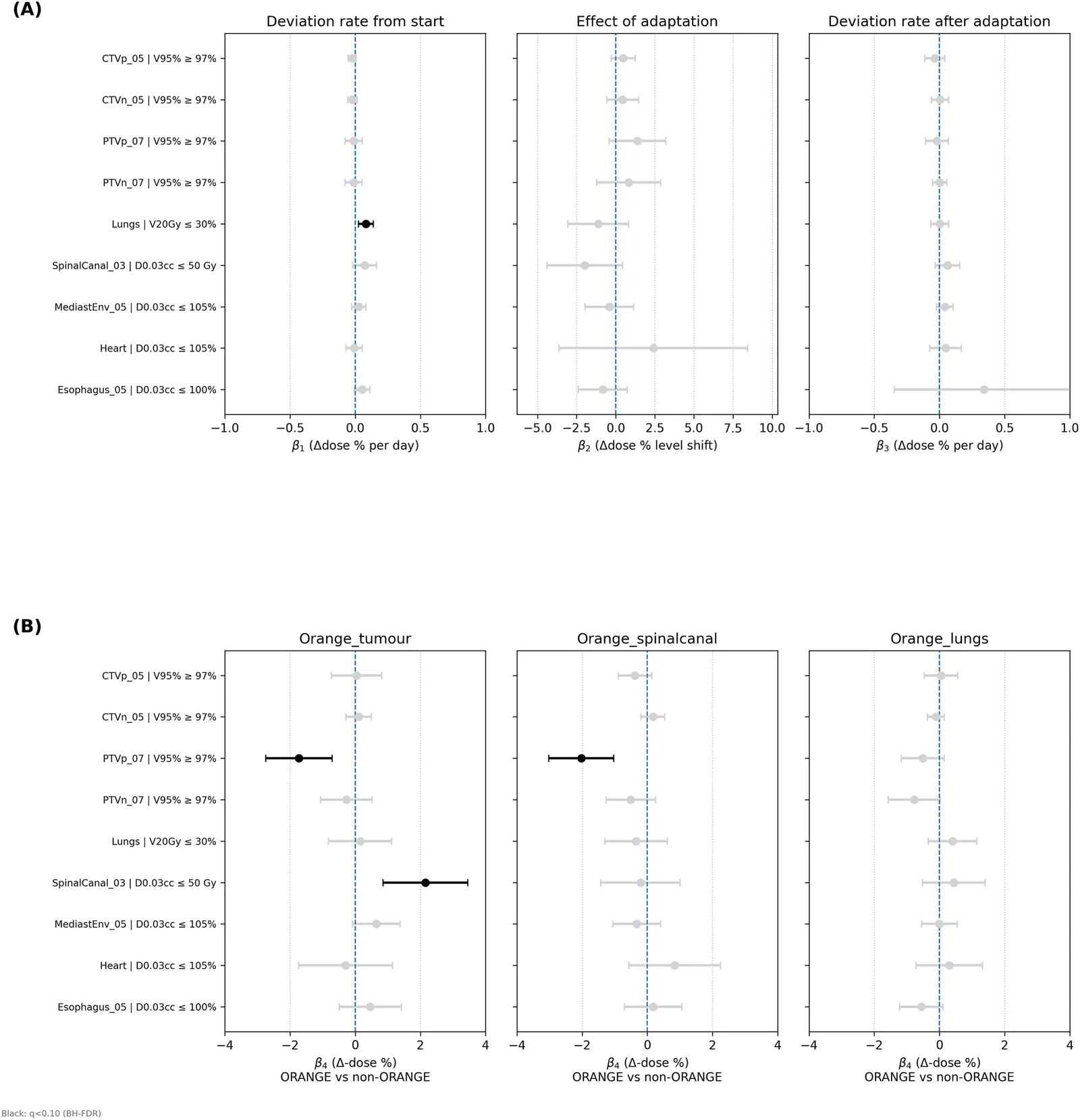
(A) Piecewise linear mixed model fixed effects for longitudinal dose deviation rates. Forest plots show the estimated fixed effect coefficients with 95% confidence intervals for the pre-adaptation slope (β₁), the immediate adaptation level shift (β₂), and the post-adaptation slope (β₃). Black markers indicate statistical significance (q < 0.10); grey markers indicate non-significant estimates. The dashed vertical line represents β = 0. (B) Association between code orange and delivered dose differences across dose constraints. Forest plots show the estimated fixed effect coefficients (β) with 95% confidence intervals for the association between code orange and fractional dose differences (Δ%). Black markers indicate q < 0.10; grey markers indicate non-significant estimates. All models included a random intercept and random slope for time, adjusted for pre-adaptation slope, level shift, and post-adaptation slope. The dashed vertical line represents β = 0.

At the population level, lung V20Gy showed a significant deviation rate before adaptation (β₁ = 0.082 percentage points of ΔV per calendar day, hereafter %-points/day; 95% CI 0.025 to 0.139; q = 0.044, Fig. 3). No other constraint reached significant deviation rate before the plan-adaptation. The effect of adaptation was directionally consistent with the intent of replanning, improving target coverage while reducing organ-at-risk dose. However, these population-level shifts did not reach significance. There was no significant post-adaptation deviation rate.

**Fig. 4:**
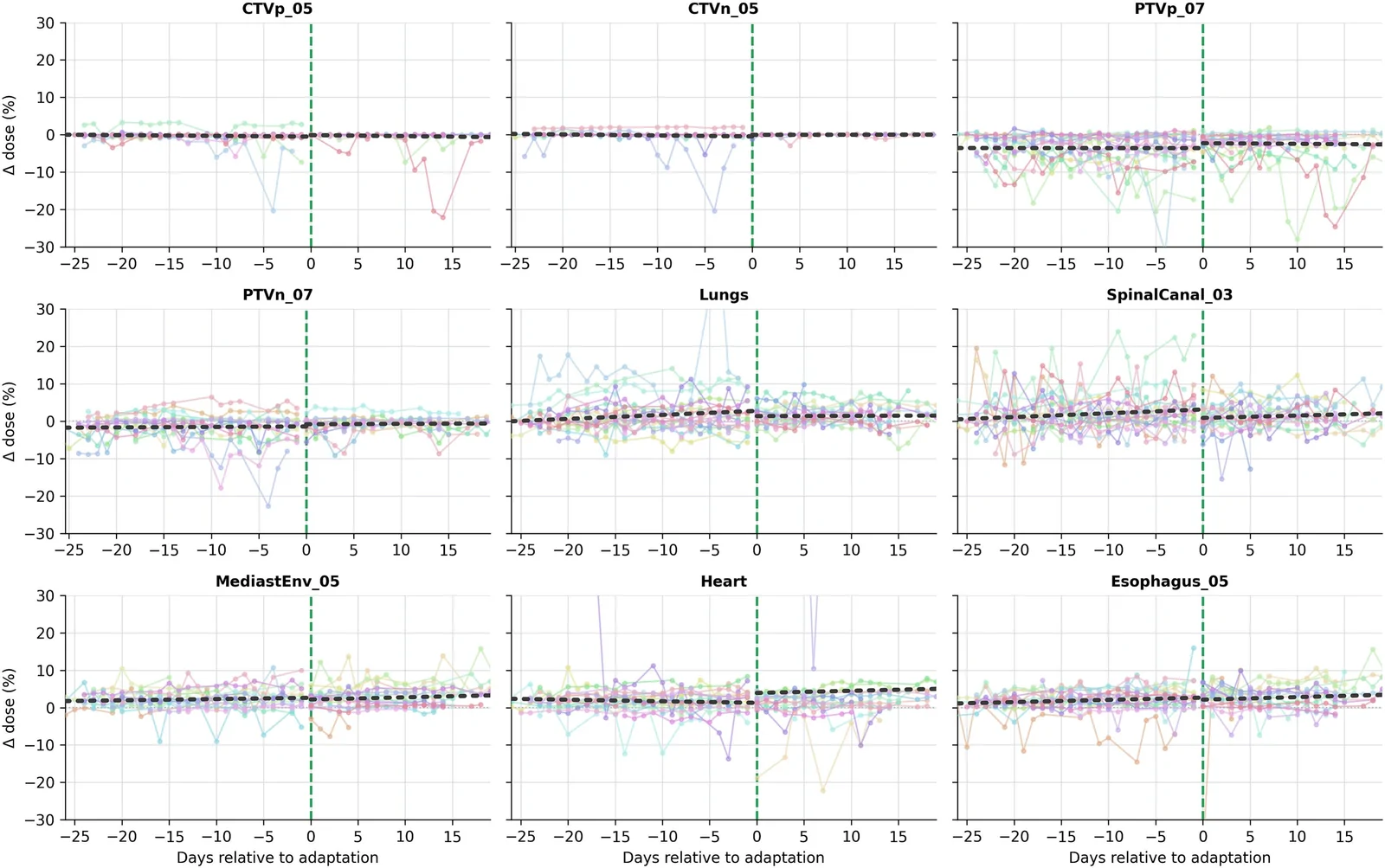
Per-patient dose constraint trajectories over days on treatment. Each coloured line represents one patient's Δ% across fractions, plotted relative to the adaptation moment (green dashed line, *t* = 0). Trajectories are displayed as piecewise segments before and after adaptation. The population deviation rate is overlaid as a thin black dashed line. (For interpretation of the references to colour in this figure legend, the reader is referred to the web version of this article.)

Descriptively fitted independent deviation rates per patient showed individual trajectories ranging from flat to drifts in either direction, both before and after adaptation, exceeding the population mean (Fig. 4, Suppl. Table 2). The CTVs were the most stable across patients. CTVp V95% deviation rates spanned from coverage loss to coverage gain: −0.33 to 0.10%-points/day before adaptation (SD0.09) and −0.52 to 0.08%-points/day afterwards (SD0.11), and CTVn V95% −0.37 to 0.02 (SD0.09) and 0.00 to 0.17 (SD0.04), respectively. The PTVs showed wider inter-patient spread. PTVp V95% ranged from −0.51 (coverage loss) to 0.30%-points/day (coverage gain) before adaptation (SD0.18) and −1.23 to 0.81 afterwards (SD0.38), and PTVn V95% from −0.41 to 0.16 (SD0.14) and −0.09 to 0.82 (SD0.21). The organ-at-risk constraints varied more across patients, with spinal canal D0.03cc the most variable: −0.30 to 0.81%-points/day before adaptation (SD0.22) and −0.90 to 1.00 afterwards (SD0.37). The esophagus deviation rate after adaptation and SD are inflated by a single extreme patient slope (9.04%-points/day; esophagus in low-dose region). Excluding it, the post-adaptation spread is comparable to the other organs-at-risk.

#### Is code orange associated with delivered dose differences?

Code orange reporting was associated with delivered dose for specific constraint-code combinations (Fig. 3). The significant associations were confined to the primary PTV and the spinal canal.

*Code orange tumour* was significantly associated with coverage loss for PTVp V95% (β_4_ = −1.73%, 95% CI −2.74 to −0.71, q = 0.011) and, among organs-at-risk, with higher spinal canal D0.03cc (β_4_ = 2.15%, 95% CI 0.85 to 3.45, q = 0.011).

*Code orange spinal canal* was significantly associated with coverage loss for PTVp V95% (β_4_ = −2.03%, 95% CI −3.02 to −1.03, q = 0.002) but showed no significant organ-at-risk association.

*Code orange lungs* showed no significant association with any constraint after adjustment for days on treatment.

Every significant association improved model fit over the longitudinal model (ΔAIC −8.0 to −14.6; LRT q ≤ 0.011). Among the non-significant combinations most showed ΔAIC near zero or positive, while two showed a modest fit improvement, PTVn V95% under code orange lungs (ΔAIC −4.2) and CTVn V95% under code orange spinal canal (ΔAIC −3.8), with low uncorrected likelihood-ratio *p*-values (0.013 and 0.016). Neither survived FDR correction on the effect estimate (q = 0.40 and 0.69) and both had confidence intervals including zero, so they were not interpreted as reliable associations (Suppl. Table 3).

## Discussion

In this prospective dose-monitoring study for LA-NSCLC, multi-task automated CBCT-based dose monitoring quantified the dose differences between plan and daily delivery over days on treatment. Per-constraint daily differences varied between patients but remained within Δ3% on average. The dominant accumulative effect was an under-coverage of the PTVp/n, while CTVp/n coverage was largely preserved. At the population level the only significant dose deviation rate was a pre-adaptation rise in lung V20Gy, while target coverage and the remaining organ-at-risk did not reach significance. This was not because effects were absent but because the per-patient deviation rates were dispersed, spanning both directions around a small mean. This is the dominant result that dose evolution over days on treatment is patient-specific, emphasizing the potential value of individualized daily dose monitoring in clinical practice.

After adjustment for longitudinal dose evolution, code orange reporting was associated with delivered-dose differences for specific combinations between code orange and dose-volume constraints rather than generally. It therefore acted as only a partial proxy for dosimetric change, predominantly identifying reductions in primary PTV coverage. Importantly, CTV coverage was largely maintained, indicating that these associations mainly reflected dose changes at the PTV margin rather than compromised clinical target coverage, consistent with the intended role of the PTV in absorbing geometric uncertainties. Tumour regression was not classified as code orange because the TLP was designed to flag enlargement or displacement that could compromise target coverage. Regression-related dosimetric changes could therefore occur during green-coded fractions, which may partly explain the limited association between code orange tumour and dose deviations. The limited number of associations should also be interpreted in light of the strict and consistent retrospective coding applied in this study. In routine clinical practice, where underreporting and, to a lesser extent, overreporting occur [7], [10], the relationship between TLP reporting and dosimetric change may be even less reliable. Although the present findings do not establish whether individual patients met criteria for adaptation, CBCT-based dose monitoring provides the objective dosimetric data required to support such decisions once clinically relevant dosimetric thresholds for ART have been defined and validated. More directly than anatomical review alone, as it does not depend on subjective image interpretation.

To our knowledge, this is the first study to combine daily CBCT-based dose monitoring with a statistical assessment of its association with anatomy-based TLP-reporting. It is especially the strength of our methodology to use mixed-effects modelling rather than a predictive (AI) approach, delivering transparent estimates with quantified uncertainty. Thomsen et al. used a comparable workflow without assessing TLP reporting; their declining CTVp coverage reversed by replanning is consistent with the directional pattern we observed, although in our cohort this decline did not reach significance [16]. The IGRT dose-monitoring trends of Suwanraksa et al. [29] (lung Dmean +0.13%/fraction, esophageal Dmean +0.11%/fraction, stable heart and cord) and Wang et al. [30], who attributed the organ-at-risk increase to rapid tumour regression, point in the same direction as our estimates, but only the lung dose rise reached significance, and the remaining trends were dominated by inter-patient variability. Methodologically, both Wang and Suwanraksa et al. relied on a DIR-based virtual-CT approach with manual contour correction across all fractions, whereas the present study employed direct dose calculation on CBCT within an automated pipeline, a distinction with implications for scalability.

More broadly, this study suggests that the methodological foundation is present for systematic, patient-specific dose monitoring within offline adaptive workflows. Compared to current patient-specific QA, measurement-based QA cannot be performed during rapid adaptive workflows, making CBCT-based dose monitoring a scalable support in repeated plan delivery measurements [31]. EPID transit dosimetry remains an interesting competing strategy, but carries important limitations: it may reveal discrepancies between anatomical change and the need for plan adaptation, but it does not provide DVH-metrics [32].

The presented framework can be utilized to investigate margin reductions [21], [33], [34], [35], [36]. The approach might shape dosimetric trigger criteria for ART in combination with (multicentric) prospective validation against clinical endpoints [37]. Beyond triggering, the observed longitudinal evolution of target coverage could inform patient-specific margin strategies. The margin could be adaptively refined based on the measured coverage trajectory, for instance using linear mixed-effects models or Bayesian approaches to derive indication- or even patient-specific margin estimates as fractions accumulate.

Some limitations should be considered. First, conventional CBCT and HyperSight acquisitions were analyzed together without explicit correction for acquisition type, since literature reported similar quality and HU-to-density conversion [19]. Future work should examine whether breathing optimized HyperSight modes could further improve dosimetric accuracy. Second, this study included a large prospectively collected dataset of 604 CBCTs coming from 22 patients. This cohort is small and should therefore be interpreted with caution for the less frequently reported codes orange (tumour and spinal canal). Additionally, all TLP codes were assigned by a single experienced observer, which ensured consistency but prevented assessment of interobserver variability. Finally, target volumes were transferred rigidly without fraction-specific editing, whereas organs at risk were propagated deformably. This conservative approach supported a scalable automated workflow but may have influenced both lung and target dose metrics. With tumour regression, the propagated lung contour may expand into regions previously occupied by tumour and include additional lung tissue within the high-dose region. At the same time, the retained PTV geometry may increasingly contain low-density or newly insufflated tissue, potentially contributing to the observed reductions in PTV coverage. Part of the target undercoverage may therefore reflect the retained original target geometry rather than underdosage of the residual tumour. These effects could not be quantified without fraction-specific target contouring. Future dose-monitoring workflows should evaluate this further [38]. For clinical implementation, a target review will be mandatory, as will the evolution towards an end-to-end level 3 dose monitoring workflow to enable wide scalability.

## Conclusion

Multi-task automated dose monitoring revealed highly patient-specific dose deviation rates across days on treatment, with population trends dominated by strong inter-patient variability. Traffic light reporting partially indicated PTV-coverage loss but was an unreliable proxy for organ-at-risk dose. Both conclusions favor individual continuous daily dose monitoring over anatomy-driven communication approaches.

## CRediT authorship contribution statement

**Dylan Callens:** Writing – review & editing, Writing – original draft, Visualization, Validation, Software, Methodology, Investigation, Formal analysis, Data curation, Conceptualization. **Emma Van Riet:** Writing – review & editing, Validation, Software, Methodology, Investigation, Formal analysis. **Jan Verstraete:** Writing – review & editing, Methodology, Conceptualization. **Patrick Berkovic:** Writing – review & editing, Methodology, Conceptualization. **Maarten Lambrecht:** Writing – review & editing, Supervision, Methodology, Conceptualization. **Wouter Crijns:** Writing – review & editing, Supervision, Methodology, Formal analysis, Conceptualization.

## Declaration of competing interest

The authors declare that they have no known competing financial interests or personal relationships that could have appeared to influence the work reported in this paper.

## Appendix A Supplementary data

Supplementary material1 Illustration of the TLP application (Suppl. Fig. 1).

Supplementary material2 Graphical visualisation of the methodology for daily dose monitoring (Suppl. Fig. 2).

Supplementary material3 Graphical visualisation of the statistical assessment (Suppl. Fig. 3).

Supplementary material4 Overview of the cohort characteristics (Suppl. Table 1).

Supplementary material5 Observed per-patient dose-deviation rates (%-points/day) (Suppl. Table 2).

Supplementary material6 Comparison between the Longitudinal and Code orange model (Suppl. Table 3). All raw data and model validations from python statsmodels and R can be shared upon reasonable request.
