# Supplementary material for: Does the traffic light protocol capture dose deviations? A longitudinal CBCT-based dose-monitoring study in adaptive RT for LA-NSCLC
Source: Tech Innov Patient Support Radiat Oncol. 2026 Aug 14;39:100427. doi: 10.1016/j.tipsro.2026.100427 (PMC13505499; doi:10.1016/j.tipsro.2026.100427)
Supplement: Supplementary file 1 [file mmc4.docx]

**Suppl. Table 1** Patient, tumour and treatment characteristics of the study cohort (n = 22).

| **Characteristic** | **n = 25** |
| --- | --- |
| **Age, years** |  |
| Median (range) | 66 (43-82) |
| Mean (SD) | 65.7 (9.1) |
| **Sex, n (%)** |  |
| Male | 19 (76.0) |
| Female | 6 (24.0) |
| **Tumour side, n (%)** |  |
| Right | 16 (64.0) |
| Left | 9 (36.0) |
| **Tumour lobe, n (%)** |  |
| Upper | 14 (56.0) |
| Lower | 9 (36.0) |
| Middle | 2 (8.0) |
| **Histology, n (%)** |  |
| Adenocarcinoma | 12 (48.0) |
| Squamous cell carcinoma | 12 (48.0) |
| Pleomorphic carcinoma | 1 (4.0) |
| **Stage (AJCC), n (%)** |  |
| IIIA | 10 (40.0) |
| IIIB | 11 (44.0) |
| IIIC | 3 (12.0) |
| IV | 1 (4.0) |
| **Treatment sequencing, n (%)** |  |
| Concurrent chemoradiotherapy | 14 (56.0) |
| Sequential chemoradiotherapy | 8 (32.0) |
| Induction | 2 (8.0) |
| Radiotherapy alone | 1 (4.0) |
| **Fractionation schedule, n (%)** |  |
| 60 Gy / 30 fr (2.0 Gy) | 11 (44.0) |
| 55 Gy / 20 fr (2.75 Gy) | 4 (16.0) |
| 66 Gy / 33 fr (2.0 Gy) | 4 (16.0) |
| 60.5 Gy / 22 fr (2.75 Gy) | 3 (12.0) |
| 45 Gy / 25 fr (1.8 Gy) | 2 (8.0) |
| 52.5 Gy / 19 fr (2.76 Gy) | 1 (4.0) |
|  |  |

*Values are n (%) unless otherwise stated. Percentages may not sum to 100 owing to rounding. AJCC, American Joint Committee on Cancer (8th edition); fr, fractions; SD, standard deviation.*
