# Supplementary material for: Does the traffic light protocol capture dose deviations? A longitudinal CBCT-based dose-monitoring study in adaptive RT for LA-NSCLC
Source: Tech Innov Patient Support Radiat Oncol. 2026 Aug 14;39:100427. doi: 10.1016/j.tipsro.2026.100427 (PMC13505499; doi:10.1016/j.tipsro.2026.100427)
Supplement: Supplementary file 2 [file mmc5.docx]

| **Suppl. Table 2** Observed per-patient dose-deviation rates (%-points/day) by structure and treatment period. Per-patient ordinary least-squares slopes were fitted on the fractional dose deviation (Δ%) versus days from treatment start, separately for the pre- and post-adaptation segments, where ≥ 3 fractions were available. n denotes the number of fitted slopes (one per patient). | | | | | | |
| --- | --- | --- | --- | --- | --- | --- |
| ***Structure / Dose constraint*** | ***Period*** | ***n*** | ***Min*** | ***Max*** | ***Mean*** | ***SD*** |
| **CTVp** V95% ≥ 97% | Pre-adaptation | 22 | -0.33 | 0.10 | -0.02 | 0.09 |
|  | Post-adaptation | 22 | -0.52 | 0.08 | -0.02 | 0.11 |
| **CTVn** V95% ≥ 97% | Pre-adaptation | 17 | -0.37 | 0.02 | -0.03 | 0.09 |
|  | Post-adaptation | 17 | 0.00 | 0.17 | 0.01 | 0.04 |
| **PTVp** V95% ≥ 97% | Pre-adaptation | 22 | -0.51 | 0.30 | 0.01 | 0.18 |
|  | Post-adaptation | 22 | -1.23 | 0.81 | 0.00 | 0.38 |
| **PTVn** V95% ≥ 97% | Pre-adaptation | 17 | -0.41 | 0.16 | 0.00 | 0.14 |
|  | Post-adaptation | 17 | -0.09 | 0.82 | 0.07 | 0.21 |
| **Lungs** V20Gy ≤ 30% | Pre-adaptation | 22 | -0.13 | 0.43 | 0.08 | 0.15 |
|  | Post-adaptation | 22 | -0.40 | 0.32 | -0.01 | 0.19 |
| **Spinal canal** D0.03cc ≤ 50 Gy | Pre-adaptation | 22 | -0.30 | 0.81 | 0.09 | 0.22 |
|  | Post-adaptation | 22 | -0.90 | 1.00 | 0.01 | 0.37 |
| **Mediastinal envelope** D0.03cc ≤ 105% | Pre-adaptation | 18 | -0.27 | 0.32 | 0.04 | 0.13 |
|  | Post-adaptation | 21 | -0.21 | 0.22 | 0.02 | 0.11 |
| **Heart** D0.03cc ≤ 105% | Pre-adaptation | 21 | -1.11 | 0.14 | -0.03 | 0.26 |
|  | Post-adaptation | 21 | -0.35 | 0.75 | 0.04 | 0.23 |
| **Esophagus** D0.03cc ≤ 100% | Pre-adaptation | 22 | -0.30 | 0.43 | 0.05 | 0.15 |
|  | Post-adaptation | 22 | -0.40 | 9.04* | 0.40 | 1.94 |
| *The esophagus post-adaptation maximum and SD are inflated by a single extreme patient slope (+9.04 %-points/day); excluding it, the post-adaptation spread is comparable to the other organs at risk.* | | | | | | |
