# Supplementary material for: Does the traffic light protocol capture dose deviations? A longitudinal CBCT-based dose-monitoring study in adaptive RT for LA-NSCLC
Source: Tech Innov Patient Support Radiat Oncol. 2026 Aug 14;39:100427. doi: 10.1016/j.tipsro.2026.100427 (PMC13505499; doi:10.1016/j.tipsro.2026.100427)
Supplement: Supplementary file 3 [file mmc6.docx]

| **Suppl. Table 3** Association between code orange and delivered dose after adjustment for longitudinal treatment evolution. For each dosimetric structure and code orange type, the fixed-effect estimate (β) from the code orange model represents the mean difference in Δ-dose (%) between flagged and non-flagged fractions. ΔAIC and likelihood ratio test p-values quantify the improvement in model fit relative to the longitudinal model. | | | | | | | | | | | | |
| --- | --- | --- | --- | --- | --- | --- | --- | --- | --- | --- | --- | --- |
|  | **Orange Tumour** | | | | **Orange Spinal Canal** | | | | **Orange Lungs** | | | |
| ***Structure / Dose constraint*** | ***β (95% CI)*** | ***q (FDR)*** | ***ΔAIC*** | ***LRT p*** | ***β (95% CI)*** | ***q (FDR)*** | ***ΔAIC*** | ***LRT p*** | ***β (95% CI)*** | ***q (FDR)*** | ***ΔAIC*** | ***LRT p*** |
| CTVp V95% | 0.04 (-0.74; 0.81) | 0.959 | 2.0 | 0.851 | -0.38 (-0.89; 0.13) | 0.493 | -0.2 | 0.140 | 0.05 (-0.47; 0.56) | 0.924 | 1.9 | 0.810 |
| CTVn V95% | 0.10 (-0.29; 0.50) | 0.821 | 44.3 | 1.000 | 0.17 (-0.20; 0.53) | 0.692 | -3.8 | 0.016 | -0.11 (-0.37; 0.15) | 0.692 | 28.9 | 1.000 |
| PTVp V95% | **-1.73 (-2.74; -0.71)** | **0.011** | **-8.7** | **0.001** | **-2.03 (-3.02; -1.03)** | **0.002** | **-14.6** | **<0.001** | -0.51 (-1.17; 0.15) | 0.493 | 0.2 | 0.176 |
| PTVn V95% | -0.27 (-1.06; 0.52) | 0.759 | 1.7 | 0.593 | -0.51 (-1.27; 0.25) | 0.565 | 0.5 | 0.222 | -0.77 (-1.57; 0.03) | 0.404 | -4.2 | 0.013 |
| Lungs V20Gy | 0.15 (-0.82; 1.12) | 0.863 | 1.9 | 0.753 | -0.34 (-1.30; 0.61) | 0.759 | 1.4 | 0.429 | 0.40 (-0.34; 1.15) | 0.692 | 0.8 | 0.272 |
| SpinalCanal_03 D0.03cc | **2.15 (0.85; 3.45)** | **0.011** | **-8.0** | **0.002** | -0.21 (-1.42; 1.00) | 0.862 | 1.9 | 0.749 | 0.44 (-0.52; 1.40) | 0.692 | 1.2 | 0.377 |
| MediastEnv_05 D0.03cc | 0.64 (-0.08; 1.37) | 0.447 | -1.0 | 0.081 | -0.32 (-1.06; 0.41) | 0.692 | 1.2 | 0.377 | 0.00 (-0.54; 0.55) | 0.995 | 2.0 | 0.950 |
| Heart D0.03cc | -0.30 (-1.74; 1.15) | 0.855 | 1.9 | 0.711 | 0.84 (-0.57; 2.24) | 0.654 | 0.6 | 0.245 | 0.30 (-0.72; 1.33) | 0.800 | 1.7 | 0.598 |
| Esophagus_05 D0.03cc | 0.46 (-0.49; 1.41) | 0.692 | 1.1 | 0.341 | 0.18 (-0.71; 1.06) | 0.855 | 1.8 | 0.696 | -0.56 (-1.23; 0.11) | 0.463 | -0.7 | 0.103 |
| *Note: The more green the cell is coloured, the better the TLP reporting fits the model for that structure based on AIC and LRT.* | | | | | | | | | | | | |
